# Genetic variation and preliminary marker-trait associations for cane quality traits in a diverse world collection of sugarcane (*Saccharum* spp.) and wild relatives

**DOI:** 10.3389/fpls.2025.1643469

**Published:** 2025-10-30

**Authors:** Shamseldeen Eltaher, Douglas DeStefano, Yasmeen Abuasbeh, Hardev S. Sandhu, Matthew Rouse, Gul Shad Ali, Sukhwinder Singh

**Affiliations:** ^1^ United States Department of Agriculture, Agricultural Research Service (USDA-ARS), Subtropical Horticulture Research Station (SHRS), Miami, FL, United States; ^2^ Department of Plant Biotechnology, Genetic Engineering and Biotechnology Research Institute (GEBRI), University of Sadat City (USC), Sadat City, Egypt; ^3^ Florida Sugar Cane League Inc., Clewiston, FL, United States; ^4^ Institute of Food and Agricultural Sciences, Everglades Research and Education Center, University of Florida, Belle Glade, FL, United States; ^5^ Sugarcane Field Station, United States Department of Agriculture, Agricultural Research Service (USDA-ARS), Canal Point, FL, United States

**Keywords:** sugarcane germplasm, marker traits association, brix, qualitative trait loci, genetics

## Abstract

**Introduction:**

Modern sugarcane cultivars originate from a limited genetic pool, primarily comprising *Saccharum spontaneum* and *S. officinarum*, which restricts yield improvements and stress resilience. It's vital to conserve and utilize genetic diversity from the World Collection of Sugarcane and Related Grasses (WCSRG) and is a key resource for future advancements.

**Methods:**

The Cane Presentation System measures essential parameters, including Brix, Polarity, moisture, and fiber content, which are critical for evaluating sugar extraction potential and processing efficiency. Molecular markers hold significant value in sugarcane breeding, and genome-wide association studies have been conducted to identify genetic loci associated with these target traits.

**Results:**

The study assessed variations in cane quality traits (Brix, polarity, fiber, and moisture content), finding that hybrids and *S. robustum* outperformed in these characters. Trait correlation analysis indicated independent genetic control, forming a basis for future research. Genome-wide association studies identified 40 significant SNPs across chromosomes 2 to 8, with markers on chromosomes 4, 6, and 7 consistently associated with Brix, and markers on chromosomes 2 and 5 linked to fiber. Additionally, marker AX-171243917-4651 on chromosome 6D was associated with both Brix and polarity.

**Discussion:**

This study examines the genetic diversity and trait associations in sugarcane, emphasizing the application of stable SNP markers linked to key characteristics, such as Brix, Polarity, and fiber content, for enhancing multiple traits through marker-assisted selection. The findings reveal significant phenotypic variation among *Saccharum* species, advocating for a broad genetic base in breeding programs. Due to sugarcane's polyploid nature, the study calls for further validation through fine mapping, gene expression analysis, and multi-location testing. Future research should enhance marker density, expand populations, and adopt new genomic approaches for effective crop improvement.

## Introduction

Sugarcane (*Saccharum* spp.) is a high-yielding perennial grass cultivated mainly for its sucrose-containing stalks. The stalks are utilized in the production of sugar and bioethanol. A significant cash crop, nearly 80% of the world’s sugar and 60% of the world’s biofuel is produced from sugarcane (Dahlquist, 2013). The genus *Saccharum* consists of several species, including *S. officinarum*, *S.* sp*ontaneum*, *S. robustum, S. sinense*, and *S. barberi*. Among them, *S. officinarum* is a high-sugar containing species with large stalks, while *S.* sp*ontaneum* is valued for characteristics such as disease resistance and tolerance to a broad spectrum of environmental conditions (Ming et al., 2006). Modern sugarcane cultivars are highly polyploid and aneuploid hybrids with 100–130 chromosomes, which are products of interspecific hybridization among these species, predominantly *S. officinarum* (2n = 80, x = 10) and *S.* sp*ontaneum* (2n = 40–128, x = 8) leading to complex genomic compositions that are difficult for genetic analysis and breeding (Hoarau et al., 2022). The initial interspecific F_1_ hybrids were backcrossed extensively to *S. officinarum* clones or other hybrids to recover high sugar content, a process named “nobilization” (Daniels and Roach, 1987; Hoarau et al., 2022).

Modern sugarcane cultivars trace back to only a few *S.* sp*ontaneum* and *S. officinarum* ancestor clones (Deren, 1995; Lima et al., 2002; Yang et al., 2020). This narrow genetic base limited sugarcane cultivar improvement for yield and tolerance to various stresses. Therefore, conservation, characterization, and utilization of genetic diversity in sugarcane germplasm collections are essential to widen the genetic base for its improvement. The World Collection of Sugarcane and Related Grasses (WCSRG), is a precious resource for future sugarcane cultivar improvement (Nayak et al., 2014; Todd et al., 2014; Yang et al., 2020). The collection comprises ∼900 accessions from 45 countries, comprising *Saccharum* germplasm and related grass species, the most frequent of which are *S.* sp*ontaneum, S. officinarum*, and interspecific hybrids. These countries, however, do not represent the center of origin of the genus. The collection holds gene resources that can potentially be used to improve yield, fiber, and abiotic and biotic stress tolerance in sugarcane breeding programs. A core germplasm collection was developed by selecting representative accessions from the WCSRG (Nayak et al., 2014; Todd et al., 2014; Yang et al., 2020), which showed sufficient natural phenotypic diversity and are valuable resources for the identification of desirable alleles controlling yield and stress tolerance for sugarcane improvement.

The Cane Presentation System (CPS) measures key parameters for harvested sugarcane that reflect its potential to meet industrial standards for sugar extraction. CPS evaluates several important characteristics of cane quality, including Brix (total soluble solids, predominantly sucrose), Polarity (proportion of sucrose in the juice), moisture (which affects processing efficiency), and fiber (affects milling ability). Brix and Polarity are key indicators of sugar yield potential, and moisture and fiber content are significant parameters for processing efficiency and biomass conversion. A precise evaluation of these characteristics is essential for maximum sugar recovery and refining breeding selection criteria in breeding programs (Jackson, 2005). Variation in sucrose content between the different *Saccharum* species is extreme and assumes a key function in sugarcane breeding programs. *Saccharum officinarum*, also known as the “Noble cane,” contains high sucrose content with a high vulnerability to disease. This species constitutes approximately 80% of the genome of modern commercial cultivars and is the principal donor of genes responsible for sucrose accumulation (Dinesh Babu et al., 2022; Khan et al., 2023). Sucrose content in sugarcane exhibits considerable diversity in different tissues of the plant and varies with the progression of plant age. Major determinants, such as genetic constitution, environmental conditions, and developmental stage, have a significant influence on sucrose accumulation in sugarcane internodes. During the growth of the plant, every internode behaves independently, and the lower internodes reach maturity first in relation to the top ones, which are in the growing phase. The plant goes through a ripening process with age, where sucrose storage is enhanced in a greater number of internodes (de Morais et al., 2015; Jackson, 2005; Khan et al., 2023).

Molecular markers have become useful tools in sugarcane breeding, significantly improving knowledge of complex genetics and assisting breeders in the genetic improvement of varieties. Their application is especially important in sugarcane breeding due to the crop’s complicated genetic makeup, high ploidy levels (ranging from 8 to 13 X), incidences of aneuploidy, variable flowering depending on environmental conditions, and a long growth period that usually exceeds a year (Wang et al., 2024). Single Nucleotide Polymorphisms (SNPs) have become the marker of choice for mapping and marker-assisted selection (MAS) in sugarcane breeding. This is not only because SNP markers are codominant, and highly abundant in the genome, but also because of the constant lowering of SNP genotyping cost and evolution of more efficient SNP genotyping techniques (Khanbo et al., 2023; Xiong et al., 2023). Over the last few years, hundreds of genetic loci and single nucleotide polymorphisms (SNPs) related to important agronomic traits in sugarcane have been uncovered using different methods, such as quantitative trait loci (QTL) mapping, gene mapping, and genome-wide association studies (GWAS) (Khanbo et al., 2023; Wang et al., 2024; Xiong et al., 2023). Genome-wide association studies (GWAS) have been utilized as a efficient tool for disassembling the genetic structure of sugarcane complex quality traits. GWAS has been particularly effective in mapping genetic variants associated with economically important traits such as Brix, polarity, moisture, fiber content, and components of yield that are critical for both sugar production as well as bioethanol conversion efficiency. Recent GWAS research has succeeded in identifying some marker-trait associations (MTAs) for significant quality traits, and studies have reported the identification of 23 MTAs for traits like soluble solid content, stalk height, stalk number, stalk weight, and cane yield (Barreto et al., 2019). Fiber and sucrose concentrations, being the two most significant quantitative traits deserving multi-year and multi-location testing, have been studied widely using GWAS approaches for creating molecular breeding strategies like marker-assisted breeding and genomic selection (Xiong et al., 2023)Identified 13 and 9 markers associated with fiber and sucrose contents, using 237 self-pollinated progenies of LCP 85-384, a popular Louisiana cultivar. Also, the application of multi-locus mixed models in GWAS has boosted the detection ability to recognize substantial associations, and top-ranked markers explained up to 15% of the overall phenotypic variation for traits of sucrose concentration. The objective of this study was to investigate (1) phenotypic diversity for cane quality traits in a diverse collection of 397 *Saccharum* accessions, (2) genotypic diversity among this diverse set of genotypes using SNP markers, and (3) marker trait associations for important cane quality traits in this diverse set of accessions.

## Materials and methods

### Plant materials

**Figure 1:**
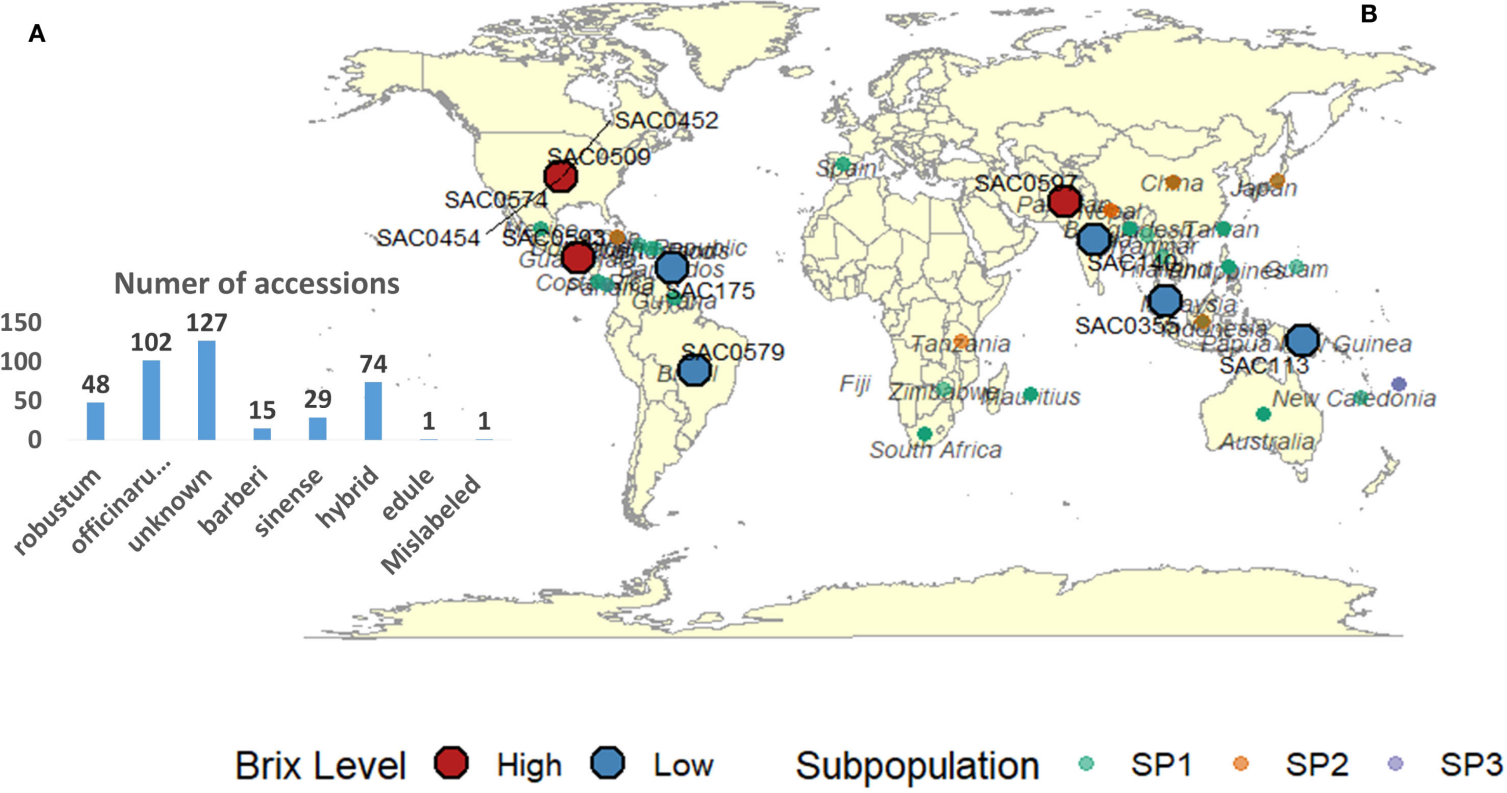
**(A)** Number of accessions per Saccharum species included in this study. **(B)** Country or institution from which each accession was obtained, as recorded in the SHRS USDA−ARS germplasm database. These records do not represent the true evolutionary or domestication origins of the 397 sugarcane accessions from the World Collection of Sugarcane and Related Grasses (WCSRG); accessions with unknown origin were excluded. Colors indicate subpopulation assignments from STRUCTURE analysis: forest green (Subpopulation 1), light red (Subpopulation 2), and light blue (Subpopulation 3). Red circles mark accessions with the highest Brix values, and blue circles mark those with the lowest.

The sugarcane accessions used in this study are maintained at the United States Department of Agriculture, Agricultural Research Service (USDA-ARS), Subtropical Horticulture Research Station (SHRS) in Miami, Florida. The number of accessions for each species, along with the country or institution from which the accession was acquired, is documented in the SHRS USDA-ARS (WCSRG) sugarcane germplasm database, as illustrated in **Figure 1**. The list of all accessions, species, country or institution from which the accession was obtained, subpopulation based on STURCURE analysis and the values of 4 cane quality traits were presented in (**Supplementary Table S1**).

### Sample collection

The cultivation of 397 sugarcane clones at the USDA-ARS Subtropical Horticulture Research Station (SHRS) in Miami, Florida, represents a significant step in our understanding of sugarcane genetics and cultivation. All accessions were planted as plant cane (first crop cycle) in single rows, following standard agronomic practices for the subtropical region of Miami. Ratoon crops were excluded from this study to reduce variability caused by environmental and physiological factors that disproportionately influence ratoon performance. Unlike plant crops, ratoon yields are highly sensitive to prior harvest conditions, soil nutrient depletion, pest pressure, and age-related decline in vigor. Including ratoon data would have confounded genotype comparisons and reduced the reliability of trait assessments across accessions. To ensure uniformity and maximize the precision of phenotypic evaluations, only plant crop data were considered (Dlamini and Zhou, 2024; Xu et al., 2021). The sugarcane stalks collected over a period spanning from June 2024 to December 2024 in five distinct batches, corresponding to the physiological maturity of each accession rather than a fixed calendar schedule. Due to the genotypic differences in growth rates and accessions’ diversity, physiological maturity was established by consistent visual and physical symptoms, including internode swelling, hardening of the rind, and yellowing of green leaves. These have been widely accepted as effective markers for sucrose accumulation and harvest preparedness in sugarcane, particularly where flowering is nil or irregular. After the removal of leaves and tops, six randomly selected stalks from each sugarcane variety were meticulously processed in the cane presentation systems (CPS). The weight of each bundle was recorded before the samples were analyzed for sucrose content using the CPS near-infrared analysis system (Bruker). The samples were then shredded with a Dedini shredder, and the resulting shredded material was processed through CPS to quantify Brix (%), juice Polarity (%), and total fiber content (%) (Legendre, (1992).

It must be understood that this study was achieved in the form of a non-replicated field trial. Replicated multi-year trials involving all 397 accessions were not possible owing due to limitations in land availability, plant materials, and finances at the Miami station, and high cost and space requirements of holding large germplasm collections. Instead, our aim was to provide extensive overview of genetic diversity and make preliminary marker–trait associations in this unique germplasm panel. These non-replicated designs have been applied to sugarcane germplasm characterization at the discovery phase with the awareness that subsequent multi-environment and replicated tests are necessary to validate marker–trait associations. Therefore, we explicitly acknowledge the limitations and have reported our results as exploratory association and diversity analyses, providing valuable hypotheses and data for breeders in addition to apparent directions for future confirmation studies.

### DNA isolation and SNP calling

As described Park et al. (2024), DNA was isolated from young harvested leaf from each sugarcane accession using BioArk leaf Kit supplied by LGC, Biosearch Technologies (https://www.biosearchtech.com/). A total of 2,000 SNPs were randomly selected from the Axiom Sugarcane 100K SNP array. Based on minor allele frequency (MAF) and linkage disequilibrium (LD) and 400 SNP were ultimately retained. Another filtering was performed using different criteria such as (1) retaining biallelic SNPs, (2) removal of SNPs with >10% missing values and (3) removal of SNPS with< 5% MAF. Finally, 357 high quality SNPs across all sugarcane accessions were used in this study. The sequences of each marker of the 357 SNPs are presented in **Supplementary Table S2**.

The chromosomal positions of the 357 SNP markers were established by aligning their sequences with the reference genome of *Saccharum* sp*ontaneum* (Nascimento et al., 2019). This species is utilized as a model for sugarcane due to its relatively high-quality genome assembly and the significant contribution of its ancestral genomes to contemporary sugarcane varieties (J. Zhang et al., 2018). *Saccharum* sp*ontaneum* comprises eight chromosomes (chr1 to chr8), and its entire genome is organized into sets of homeologous chromosomes identified as A, B, and D. These sets reflect the polyploid and aneuploid nature of sugarcane, a key aspect of the species’ genetic makeup.

### The statistical analysis

A one-way analysis of variance (ANOVA) was conducted to estimate the genetic variability among the sugarcane accessions for the cane quality traits (Brix, polarity, moisture and fiber). Since our data included unreplicated genotypic observation, the analysis was done based on the genotype values of each trait and the genetic variability was tested among the subpopulation resulted from the structure finding. The model used for this analysis is expressed as follows.


$$F=\frac{\sum \left[n_{i}(\mu_{i}-\mu {)}^{2}\right]}{K-1}÷\frac{\sum \sum (x_{ij}-\mu_{i}{)}^{2}}{n-K}$$

where F is the calculated F-statistic, Σ is summation, n_i = sample size in subpopulation I, μ = overall mean, μ_i = mean of group I, x_ij = j-th observation in subpopulation I, K = number of subpopulation and n = total number of observations.

Pearson’s correlation analyses were performed among these traits, and the resultant correlation coefficients (*r* values) were calculated using the “cormat” function in R-4.4.1 (Team, R. C et al., 2016) software and the upper triangle heatmap was visualized using the “ggplot2” package (Wickham, 2016). The genetic distance analysis was performed using PowerMarker software V 3.25 (Liu and Muse, 2005) and the dendrogram cluster was visualized using MEGA 11 (Tamura et al., 2021).

### Analysis of population structure

A model-based (Bayesian) method with the 357 SNPs was utilized to evaluate the possible number of subpopulations in the accessions used in this study. The analysis of population structure was performed using STRUCTURE 3.4.0 (Pritchard et al., 2000). Structure was analyzed by means of k-values (an assumed fixed number of subpopulations K) from 1 to 10 in each K. For each K, three independent runs were performed using an admixture ancestry model with correlated allele frequencies. Each run included 50,000 burn-in iterations followed by 100,000 Markov Chain Monte Carlo (MCMC) replications and sampling frequency every 1,000 reps. These parameters were chosen based on the protocol of (Park et al., 2024). The optimal number of model components (K) was determined based on delta k (Evanno et al., 2005). The best k for the current population was determined using STRUCTURE SELECTOR (Li and Liu, 2018). The analysis of molecular variance (AMOVA) and the calculation of fixation index were conducted as described before (Peakall and Smouse, 2006). All allelic diversity pattern including number of private allele (PAL), number of different allele (Na), number of effective allele (Ne), number of common allele (N_com_) and the Shannon information diversity index (H) were calculated using R 4.41 (Team, R. C et al., 2016).

### Genome-wide association study for cane quality traits

Genome-wide association studies (GWAS) were conducted for all traits utilizing the rMVP R package (Yin et al., 2021). This analysis employed three distinct GWAS models: (1) the Fixed and Random Model Circulation Probability Unification (FarmCPU), (2) the Generalized Linear Model (GLM), and (3) the Mixed Linear Model (MLM). To address population structuring and kinship (Kin), principal component analysis (PCA) and PCA combined with Kin were incorporated into FarmCPU and MLM each individually. FarmCPU integrates the advantages of mixed linear models and stepwise regression (fixed effect models), employing them in an iterative manner to adjust for balancing population structure and overfitting the model (Liu X. et al., 2016).

## Results

### The descriptive analysis for cane quality traits

Polarity, Brix, fiber and moisture analyses were conducted using CPS near infrared to evaluate sugarcane accessions. A summary of these analyses is presented in **Table 1**. The Brix values ranged from 1.13 to 21.96 with a mean of 14.83 and a standard deviation of 3.32. Polarity had a mean of 46.07 and SD = 16.00, with values ranging from 2.42 to 83.54. The mean moisture content was 61.38 and SD = 19.20, and the median was 69.77, indicating a left-skewed distribution because the mean was lower than the median. Fiber showed moderate variability, with a mean of 17.23, SD = 4.92 and a range of 2.22 to 42.03.

**Table 1:** Descriptive statistics for the cane traits of *Saccharum* species, including brix, polarity, moisture, and fiber content.

Cane Quality Traits	Mean	SD^*^	Median	Min^*^	Max^*^	Range	SE^*^
Brix	14.83	3.32	15.42	1.13	21.96	20.83	0.16
Polarity	46.07	16.00	47.9	2.42	83.54	81.12	0.80
Moisture	61.38	19.20	69.77	7.14	79.8	72.66	0.96
Fiber	17.23	4.91	16.76	2.22	42.03	39.81	0.24

^*^SD, stander deviation; min, minimum; max, maximum and SE, stander error.

**Figure 2:**
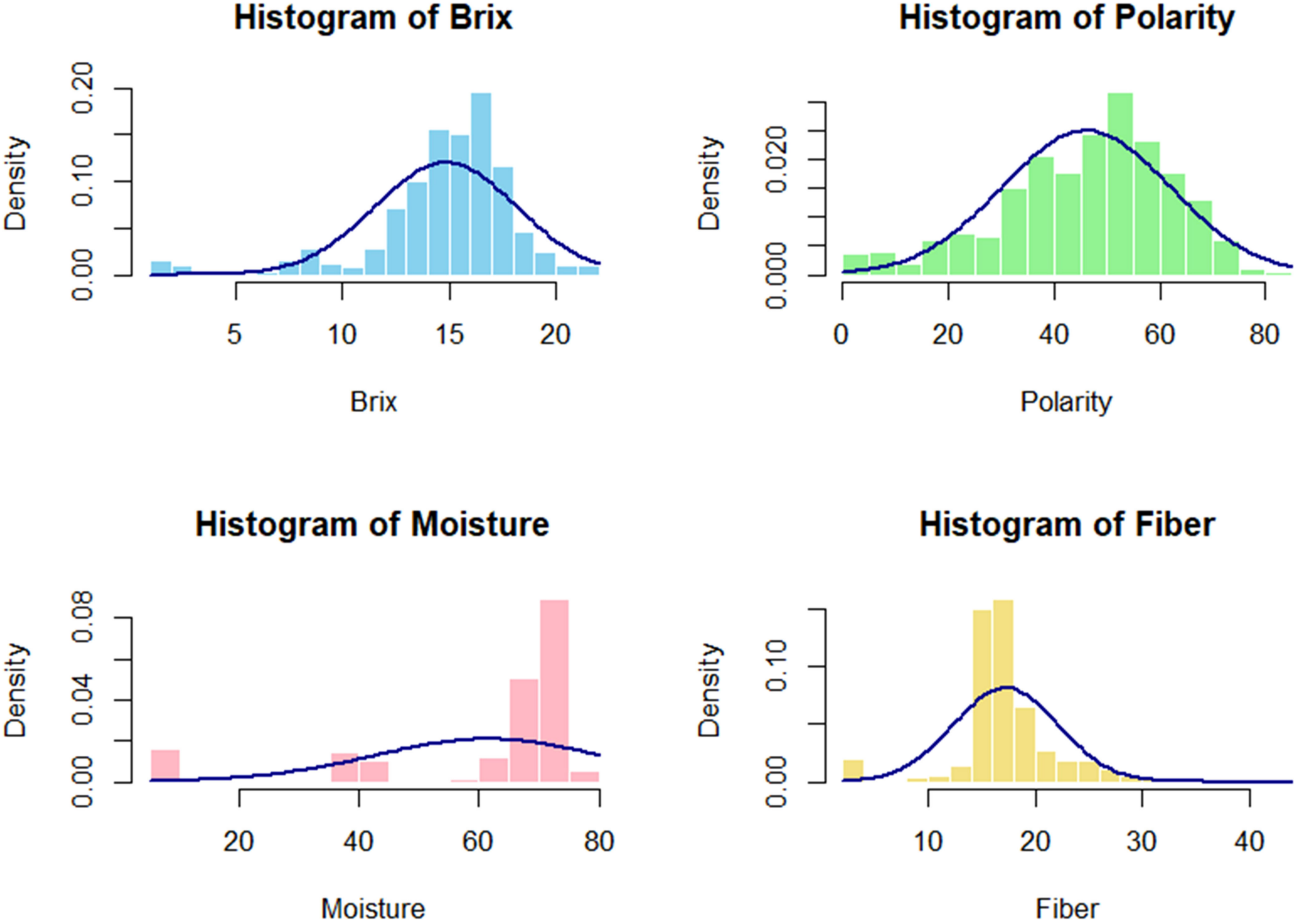
The phenotypic distribution of cane quality traits in the 397 sugarcane accessions exhibited a visually normal frequency distribution for all characteristics, with some showing skewness to the right and left.

The distributions of various traits within the germplasm collection provide significant insights into their variability, as illustrated in **Figure 2**. The histogram for Brix and Polarity reveals a bell-shaped normal distribution, indicating that most sugarcane samples exhibit moderate to high sugar content and a wide variation in sugar levels. The germplasm is generally well-suited for sugar production, as most samples exhibit moderate Brix values, with only a few showing extremely low or high levels. Conversely, the moisture histogram illustrates a left-skewed distribution, reflecting a predominance of samples with elevated moisture content.

**Figure 3:**
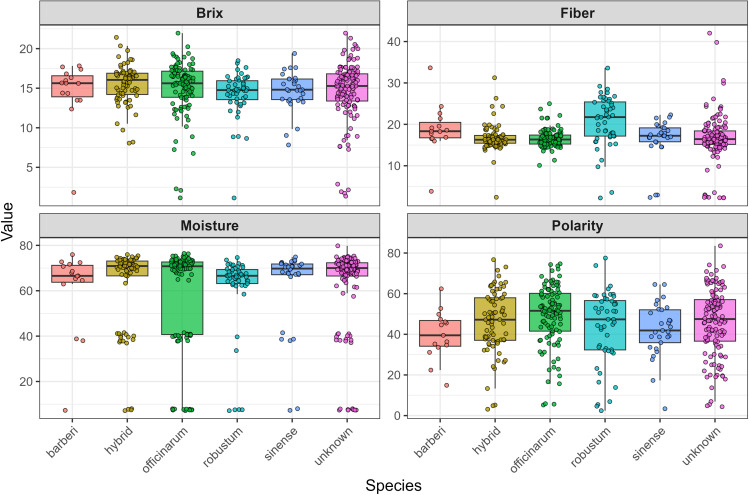
Box plots illustrating the distribution of Brix, Polarity, Moisture, and Fiber for *Saccharum* ssp. Accessions after removing one genotype mislabeled and one S. edule species.

**Figure 3** illustrates box plots that depict the distribution of four essential cane quality traits (Brix, Fiber, Moisture, and Polarity) across six accessions of *Saccharum* species, after excluding one incorrectly labeled genotype and one *S. edule* species from the dataset. The data uncovers significant patterns in trait variability that are relevant for sugarcane breeding and processing applications. Upon examining the distributions, the Brix measurements indicate relatively stable performance across most species, with medians clustering around 15–17 units and comparable interquartile ranges. However, contrary to initial expectations, the hybrid species do not consistently exhibit the highest values across all traits. In terms of fiber content, *S. robustum* stands out with the highest median values, approximately 25 units, which significantly exceeds other species that range between 15–20 units. The moisture content displays the most distinct variation, with *S. officinarum* showing an exceptionally wide distribution from nearly 0 to about 80 units, while other species maintain more uniform levels around 65–75 units. Polarity measurements reveal moderate variation among species, with most distributions centered between 40–60 units, yet exhibiting different spreads and notable outliers. These trait distributions highlight considerable variability both within and among species, offering valuable insights for breeding programs aimed at enhancing agricultural practices and formulating processing strategies. The intricate patterns observed suggest that various species may provide unique advantages for specific applications, with *S. robustum* demonstrating potential for high-fiber applications and the necessity for careful selection within the *officinarum* group due to its significant moisture variability.

### Correlation coefficient analysis

**Figure 4:**
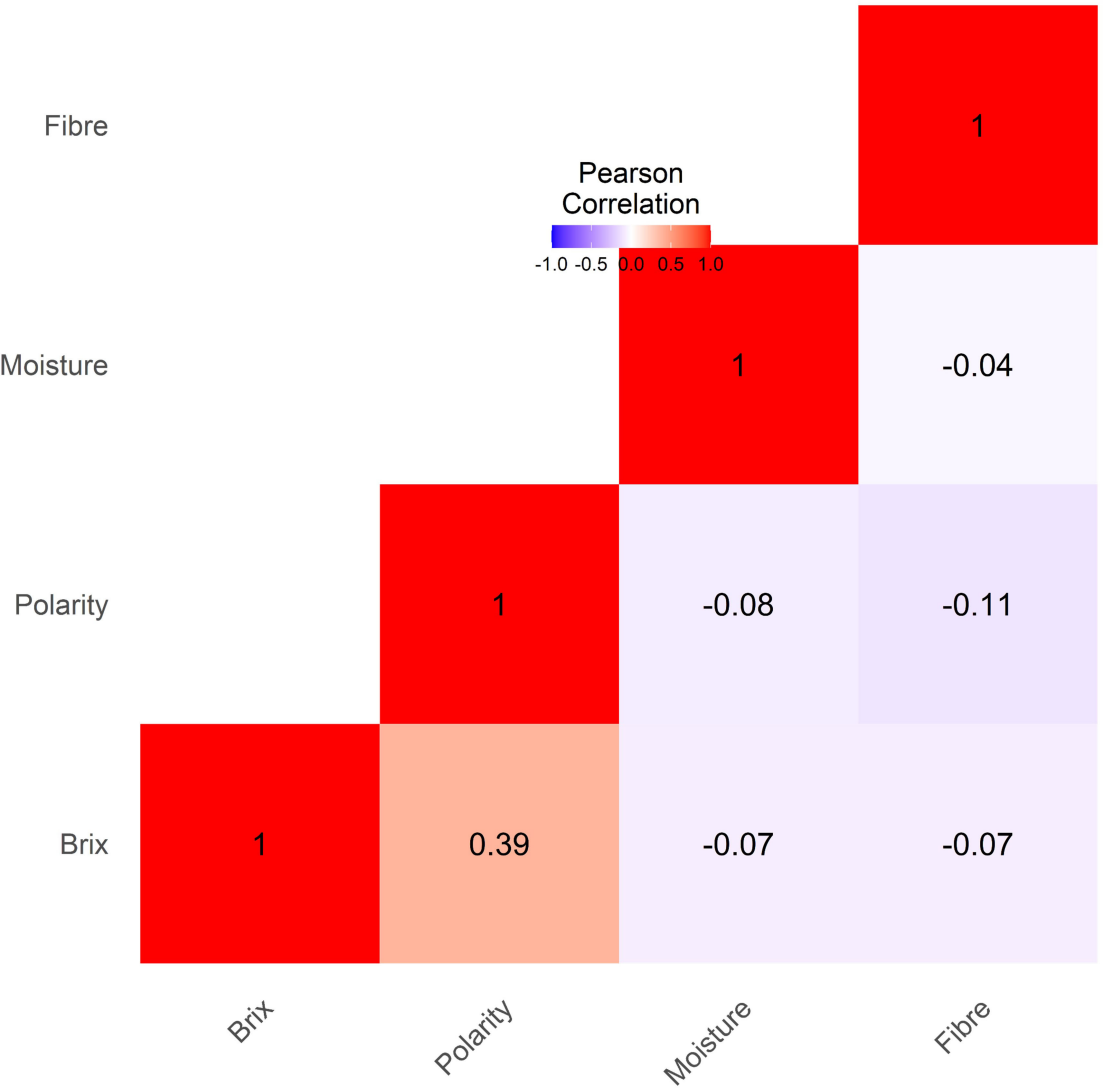
Phenotypic correlation analysis among the cane traits (Brix, Polarity, Moisture and Fiber) showed the high correlation between Brix and Polarity.

The Pearson correlation matrix illustrates the interrelationships among Brix, polarity, moisture, and fiber as depicted in **Figure 4**. A moderate positive correlation (r= 0.39***) indicates that an increase in Brix is associated with a slight increase in Polarity. On the other hand, a weak negative and non-significant correlation was observed between the remaining traits.

### Population structure and relationships

**Figure 5:**
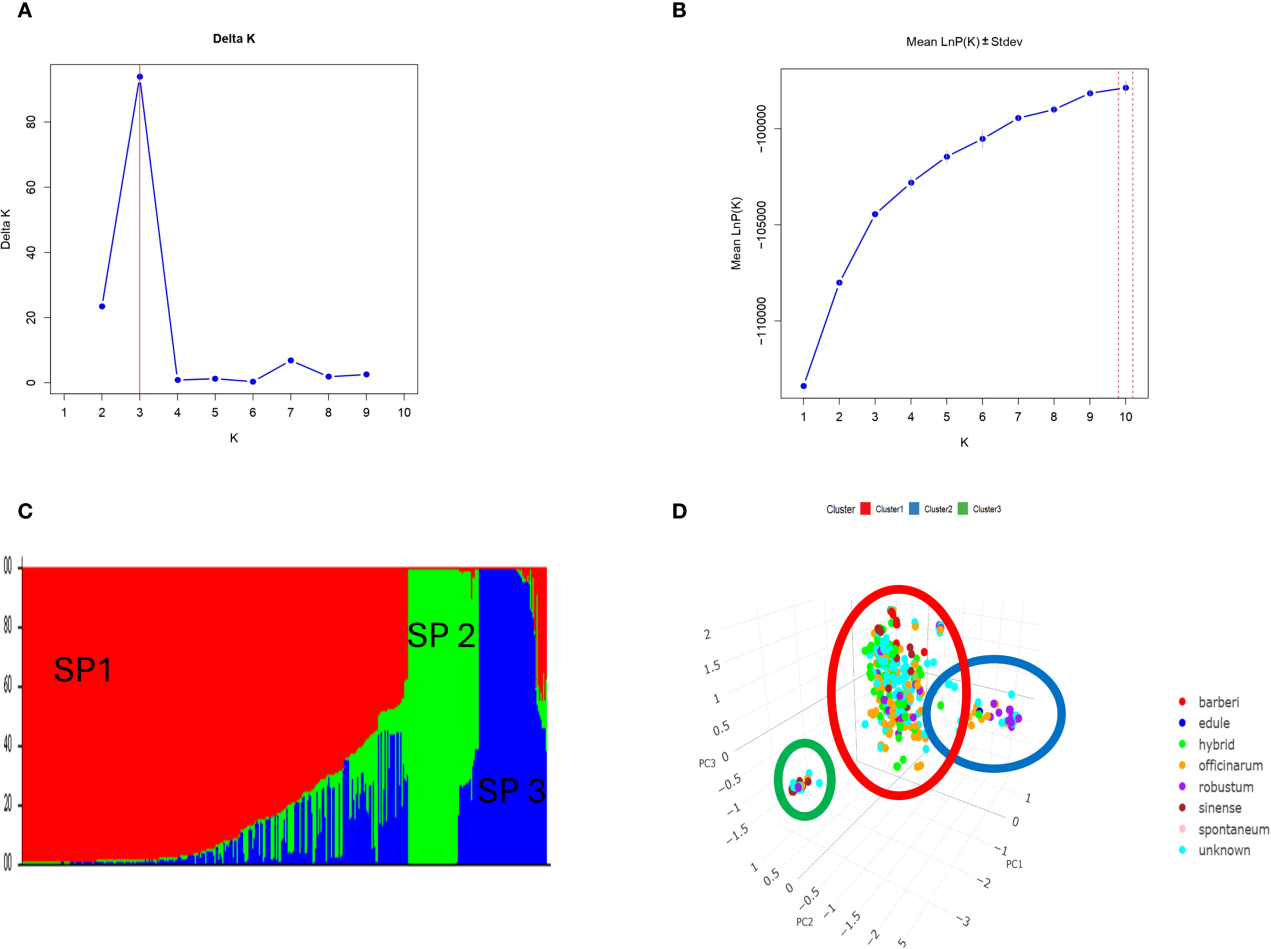
**(A)** Delta (Δ)K for differing numbers of subpopulations (k), **(B)** the average of log-likelihood value and **(C)** estimated population structure of 397 *Saccharum ssp*. on (k = 3), SP refers to different subpopulation using STRUCTURE and **(D)** Principal component analysis (PCA) based on genetic distance (SNPs) supported the STURCTURE results.

The population structure of 397 sugarcane genotypes was examined with the STRUCTURE analysis software (**Figure 5**). To determine the fitting value of the best K, the number of clusters (K) was plotted against ΔK, revealing a strong peak at k = 3 (**Figure 5A**). However, the log likelihood [LnP(D)] increased continuously and gradually as K increased (**Figure 5B**). The optimal K value was 3, suggesting that the 397 sugarcane genotypes can be grouped into three subpopulations (SP). With 292 belonging to SP1, 53 to SP2, and 52 to SP3 subgroups (**Figure 5C**). The results of PCA were consistent with STRUCTURE analyses (**Figure 5D**).

### Analysis of variance among subpopulations identified by population structure

The one-way ANOVA results are presented in **Table 2**, revealing significant differences among subpopulations defined by the population structure analysis. Polarity and fiber content showed highly significant variation (F = 17.27, *p* = 3.97 × 10^-5^; and F = 34.73, *p* = 8.12 × 10^-9^, respectively). In contrast, Brix and moisture content did not differ significantly among subpopulations (F = 2.208, *p* = 0.138; and F = 0.949, *p* = 0.33, respectively), indicating that these traits were relatively consistent across groups.

**Table 2:** The analysis of variance (ANOVA) conducted among subpopulations identified by population structure for the cane quality traits of Brix, polarity, moisture, and fiber.

Trait	Source of Variance	Df	Sum Sq	Mean Sq	F value	Pr(>F)
Brix	Subpopulation	2	24	24.38	2.208	0.138ns
	Residuals	394	4361	11.04		
Polarity	Subpopulation	2	4252	4252	17.27	3.97E-05 ***
	Residuals	394	97224	246		
Moisture	Subpopulation	2	350	350	0.949	0.33ns
	Residuals	394	145634	368.7		
Fiber	Subpopulation	2	774	774.3	34.73	8.12E-09***
	Residuals	394	8807	22.3		

ns refer to nonsignificant and *** Significant at *p*-value < 0.001.

### Comprehensive assessment of genetic diversity and differentiation in the WCSRG collection

Subpopulations showed considerable genetic divergence, and the average distance (expected heterozygosity) of each subpopulation is shown in **Table 3**. With an average of 0.26, SP1 had the highest expected heterozygosity, while SP2 had moderate value at 0.21 and SP3 had the lowest at 0.11. To examine genetic differentiation due to population substructure, the Fixation index (F_st_) was computed, which is good indicator for evaluating total genetic variation among subpopulations. The F_st_ values for SP1, SP3, and SP3 were 0.06, 0.46, and 0.56, respectively. Utilizing the three subpopulations found in the STRUCTURE study, the AMOVA, and number of migrant (Nm) were computed, which are presented in **Table 4**. Approximately 99% of the variation was observed across individuals with only 1% occurring among subpopulations. The high haploid Nm value (29.66) suggests significant historical gene exchange and genetic connectivity among species or groups, based on allele frequency patterns. These findings showed that there was considerable genetic differentiation within subpopulations and low genetic differentiation between subpopulations.

**Table 3:** The STRUCTURE analysis results of 397 sugarcane accessions included the fixation index (*F_st_*), average distances (expected heterozygosity), and the number of genotypes in each subpopulation.

Subpopulation	Fst^1^	Ex. Hetro^2^	No. of genotypes
SP1	0.06	0.26	292
SP2	0.46	0.21	53
SP3	0.56	0.11	52

^1^F_st_ is a measure of genetic differentiation among three subpopulations: ^2^Expected Heterozygosity.

**Table 4:** Analysis of molecular variance (AMOVA) for 397 sugarcane accessions grouped into three clusters.

Source	df	SS	MS	Est. Var.	%
Among Subpopulations	2	393.787	196.894	0.485	1%
Among Individuals	394	45379.996	115.178	57.589	99%
Within Individuals	397	0.000	0.000	0.000	0%
Total	793	45773.783		58.074	100%
Nm	29.66				

Nm refers to gene exchange rate or number haploid migrants .

### Allelic diversity pattern across *Saccharum* species

To further evaluate species-specific genetic contributions in the sugarcane germplasm panel, we calculated some parameters of allelic diversity, including the number of private alleles (PAL), number of different alleles (Na), number of common alleles (N_com_), number of effective alleles (Ne), and Shannon’s diversity index (H). Among the species, *S. robustum, S. officinarum*, and *S. barberi* possessed the maximum number of private alleles (3, 2, and 3, respectively) followed by hybrid and unknown types, each of which carried very few private alleles. These species also indicated higher average values of Na, Ne, and H, which indicate a broader allelic range. On the other hand, *S*. *sinense*, *S*. *edule*, and the mislabeled accession which had no private allele and had the least allelic diversity (Na = 1, Ne = 1, H ≈ 0. These findings provide a quantitative overview of the genetic distinctness and diversity retained in each species group. The values for the complete allelic diversity pattern are given in **Table 5**. The WCSRG germplasm collection, which includes accessions obtained from 36 countries crossing diverse geographical regions across the globe, reflects this complexity. The broad representation enhances our ability to detect meaningful patterns of genetic differentiation and provides a strong foundation for future breeding strategies aimed at improving sugarcane performance under varied environmental conditions.

**Table 5:** Mean of allelic diversity pattern including number of private allele (PAL), number of different alleles (Na), number of common allele (N_com_), number of effective allele (Ne) and Shannon’s index (H), in each *Saccharum* species.

Species	PAL	Na	N_com_	Ne	H
*officinarum*	2	2.02	2.02	1.52	0.49
*unknown*	2	2.04	2.04	1.52	0.49
*hybrid*	1	1.99	1.99	1.54	0.48
*robustum*	3	2.01	2.01	1.40	0.40
*barberi*	3	1.79	1.79	1.38	0.36
*sinense*	0	1.79	1.79	1.32	0.33
*edule*	0	1	1	1	-1E-10
Mislabeled	0	1	1	1	-1E-10

### Genome-wide association study of cane traits

**Figure 6:**
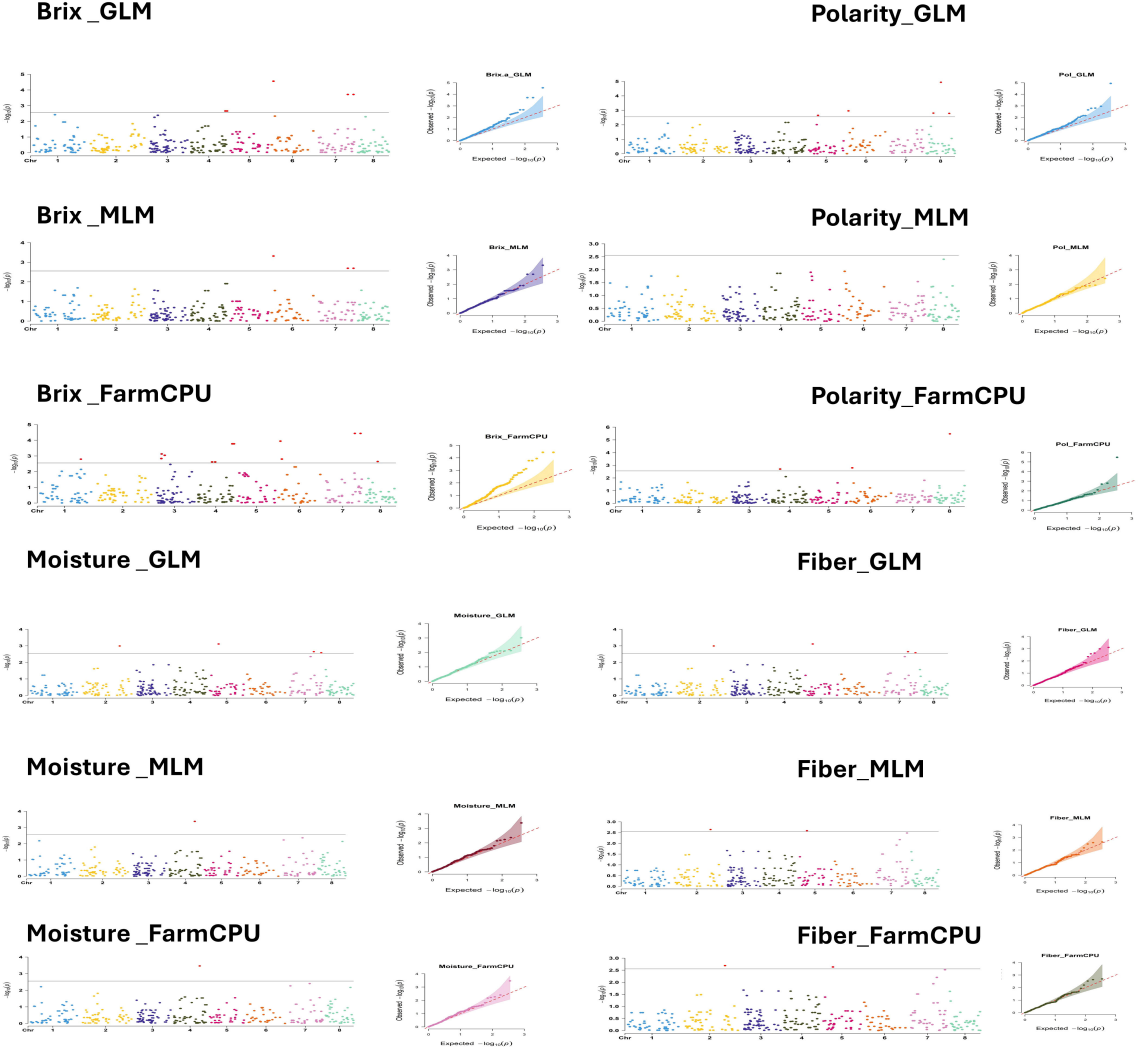
Manhattan plots displaying SNP marker-trait association identified for Brix, polarity, moisture and fiber content using GLM, MLM and FarmCPU GWAS model with 357 SNPs markers.

Marker-trait associations were investigated using three different GWAS models with 357 SNP markers. These analyses revealed 15, 6 and 19 significant SNPs markers using GLM, MLM and FarmCPU models, respectively (**Supplementary Table S3**). Significant markers were associated with Brix, fiber and moisture in GLM, MLM and FarmCPU. Significant markers were found for Polarity in two of the three models. The Manhattan and Q-Q plots for the significant traits of Brix, polarity, moisture and fiber are illustrated in **Figure 6**. For Brix, 21 markers were found significant in all GWAS models. All markers were in chromosomes 3, 4, 6 and 7. Three markers (AX-171243917-4651; chr. 6D, AX-171305424-5045; chr.7B and AX-171305424-5356; chr. 7D) were found common in the three GWAS models and two markers (AX-171270569-3055; chr.4B and AX-171270569-3274; chr.4C) were supported with GLM and MLM. The GLM and FarmCPU also identified 8 significant markers linked to polarity, distributed across chromosomes 4, 5, 6 and 8. Two markers (AX-171243917-4651; chr. 6D and AX-171247985-5866; chr. 8D) were confirmed with GLM and FarmCPU. One marker AX-171269335-3422; chr.4D was found significantly associated with moisture and supported with the three GWAS models. Eight markers were significantly associated with the fiber content and all these markers resided in three different chromosomes 2, 5 and 7. Two markers (AX-171287224-1157; chr. 2A and AX-171363600-3972; chr. 5D) were validated with three GWAS models. All the common markers in the GWAS models are presented in **Supplementary Table S4**.

### Allelic effects and their relationship to known sucrose QTLs

**Figure 7:**
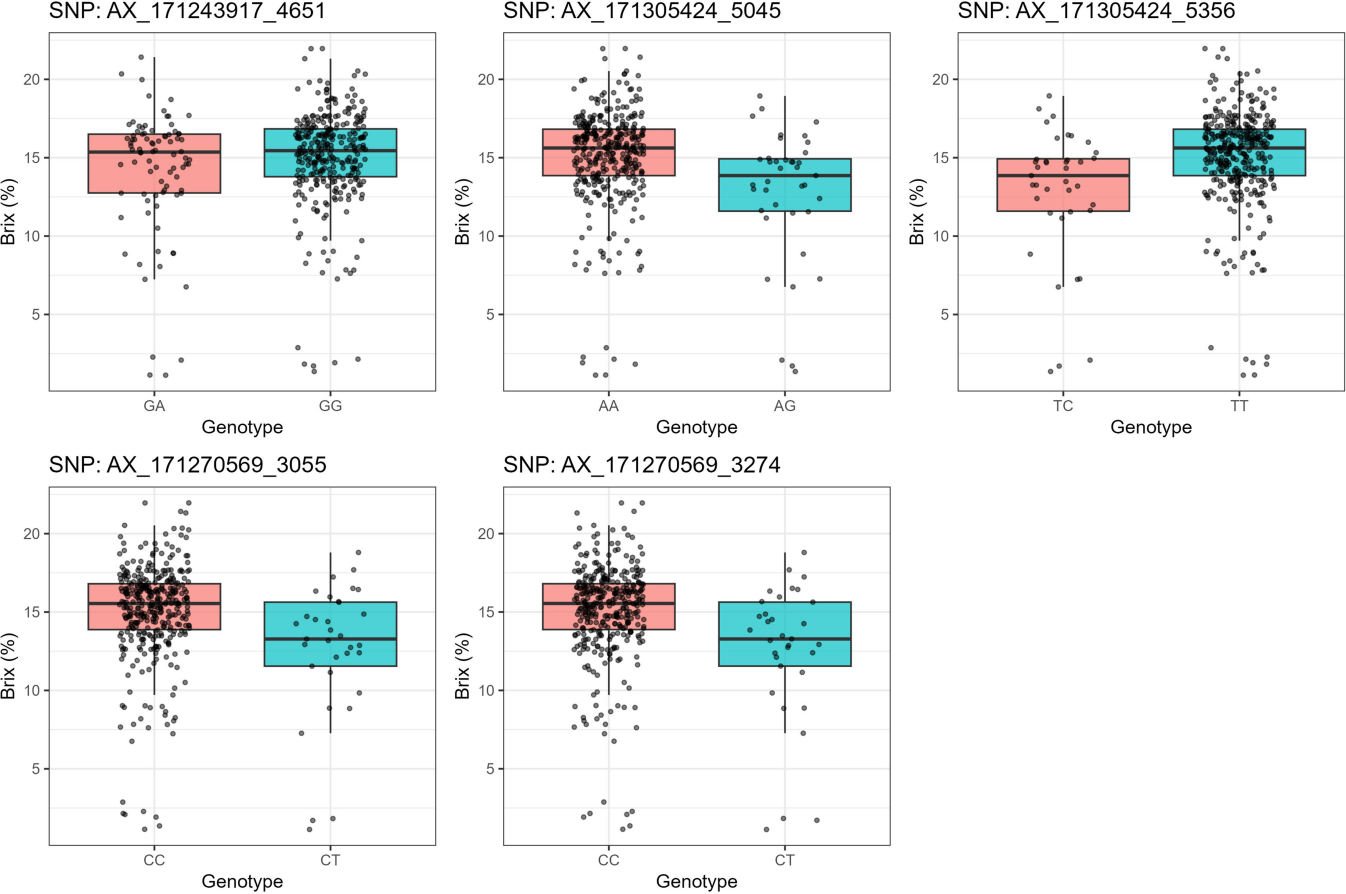
Box plots showing the difference between 397 sugarcane accessions with the target allele and without the target allele for 5 significant markers associated with Brix.

To additionally validate the associations uncovered by the GWAS, we examined the allelic effects of five top-ranked SNPs on Brix values (**Figure 7**; **Table 6**). Significant associations were identified between Brix content and several SNP loci. For AX-171243917-4651 (Chr6D:4287042–4287343), the GA genotype had a mean Brix of 14.15 ± 0.43 (SE) compared with 15.03 ± 0.18 (SE) for GG (*p* = 0.0296). At AX-171305424-5045 (Chr7B:80181102–80181403), the AA genotype showed a higher mean Brix of 15.06 ± 0.17 compared with 12.76 ± 0.68 for AG (*p* = 3.26 × 10^-5^). A similar pattern was observed for AX-171305424-5356 (Chr7D:67813880–67814181), where TT exhibited a higher Brix (15.06 ± 0.17) relative to TC (12.76 ± 0.68) with the same significance level (*p* = 3.26 × 10^-5^). For AX-171270569-3055 (Chr4B:75471756–75472057), the CC genotype had a mean Brix of 15.05 ± 0.16 compared with 12.55 ± 0.76 for CT (*p* = 3.06 × 10^-5^). Likewise, AX-171270569-3274 (Chr4C:79075825–79076126) showed higher Brix in CC (15.05 ± 0.16) compared with CT (12.55 ± 0.76), with a similar level of significance (*p* = 3.06 × 10^-5^). Overall, these results indicate that favorable homozygous genotypes consistently conferred higher Brix values than their heterozygous counterparts, suggesting strong marker–trait associations for sugar content in sugarcane.

**Table 6:** Genotypic grouping and statistical association of significant SNPs with Brix content across homozygous and heterozygous alleles.

Significant SNP	Genotype	Mean_Brix	SD_Brix	SE_Brix	No. of genotypes	*P*_ value
AX-171243917-4651: Chr6D:4287042-4287343	GA	14.15	3.99	0.43	86	0.0296
	GG	15.03	3.08	0.18	309	0.0296
AX-171305424-5045: Chr7B:80181102-80181403	AA	15.06	3.11	0.17	356	3.26E-05
	AG	12.76	4.27	0.68	39	3.26E-05
AX-171305424-5356: Chr7D:67813880-67814181	TC	12.76	4.27	0.68	39	3.26E-05
	TT	15.06	3.11	0.17	356	3.26E-05
AX-171270569-3055: Chr4B:75471756-75472057	CC	15.05	3.12	0.16	362	3.06E-05
	CT	12.55	4.38	0.76	33	3.06E-05
AX-171270569-3274: Chr4C:79075825-79076126	CC	15.05	3.12	0.16	362	3.06E-05
	CT	12.55	4.38	0.76	33	3.06E-05

### Identification of promising accessions for sugarcane breeding programs based on brix content

Selection of the 10 highest and 10 lowest sugarcane accessions, based on Brix values, holds great promise for sugarcane breeding programs. **Figure 1**, depicting the global distribution map of the selected sugarcane accessions, is a testament to this potential due to most high Brix values accessions belonging to tropical and subtropical regions. The Brix values for the highest group ranged from 19.91 to 21.96, while the lowest group exhibited values between 1.13 and 2.88. These extremes underscore the significant phenotypic diversity within the WCSRG germplasm, a diversity that we are poised to harness for the betterment of sugarcane breeding programs (**Supplementary Table S5**).

Among the accessions with the highest Brix values, two notable clones are “SAC0454” (belonging to *S. officinarum*, of US, SP3) and “SAC0168” (considered an unknown in both species and country, SP1), both of which recorded a Brix content of 21.96. These accessions represent ideal candidates for direct selection or use as parental lines in hybridization programs. Moreover, noteworthy Brix values of 21.42 and 20.35 were recorded in “SAC0593” (SP1, a hybrid from Guatemala) and “SAC0452” (a hybrid from the US), respectively. This observation indicates that accessions with hybrid backgrounds, particularly those from Guatemala and US breeding programs, may possess allelic advantages for high sugar accumulation due to their selection history and adaptation to tropical and subtropical environments. Notably, eight of the 10 highest Brix accessions were classified under SP1, suggesting that this group is particularly enriched with beneficial alleles for high sucrose content. Additionally, six top-performing accessions were categorized as unknown species, which raises the possibility that they may represent hybrids or belong to *S. officinarum*. Hybrid backgrounds may indicate genetic exchange with *S. officinarum*, *S.* sp*ontaneum*, or *S. robustum*, potentially contributing to improved Brix values.

Conversely, the lowest Brix accessions, specifically “SAC0175” (belonging to *S. robustum*, from Barbados, with a Brix of 1.13) and “SAC0113” (belonging to *S. officinarum*, from Papua New Guinea, with a Brix of 1.14), were also found within SP1. These accessions may represent unselected or ancestral lines with reduced sucrose accumulation capabilities. Nevertheless, low Brix content accessions could retain value for traits such as stress resistance, biomass production, and disease resistance qualities that are typically associated with wild *Saccharum* species preserved in ancestral collections. Importantly, the countries or institutions listed for each accession refer not to their geographic origin, but to the source from which the accession was obtained, as recorded in the Subtropical Horticulture Research Station (SHRS) at the USDA-ARS germplasm database.

**Figure 8:**
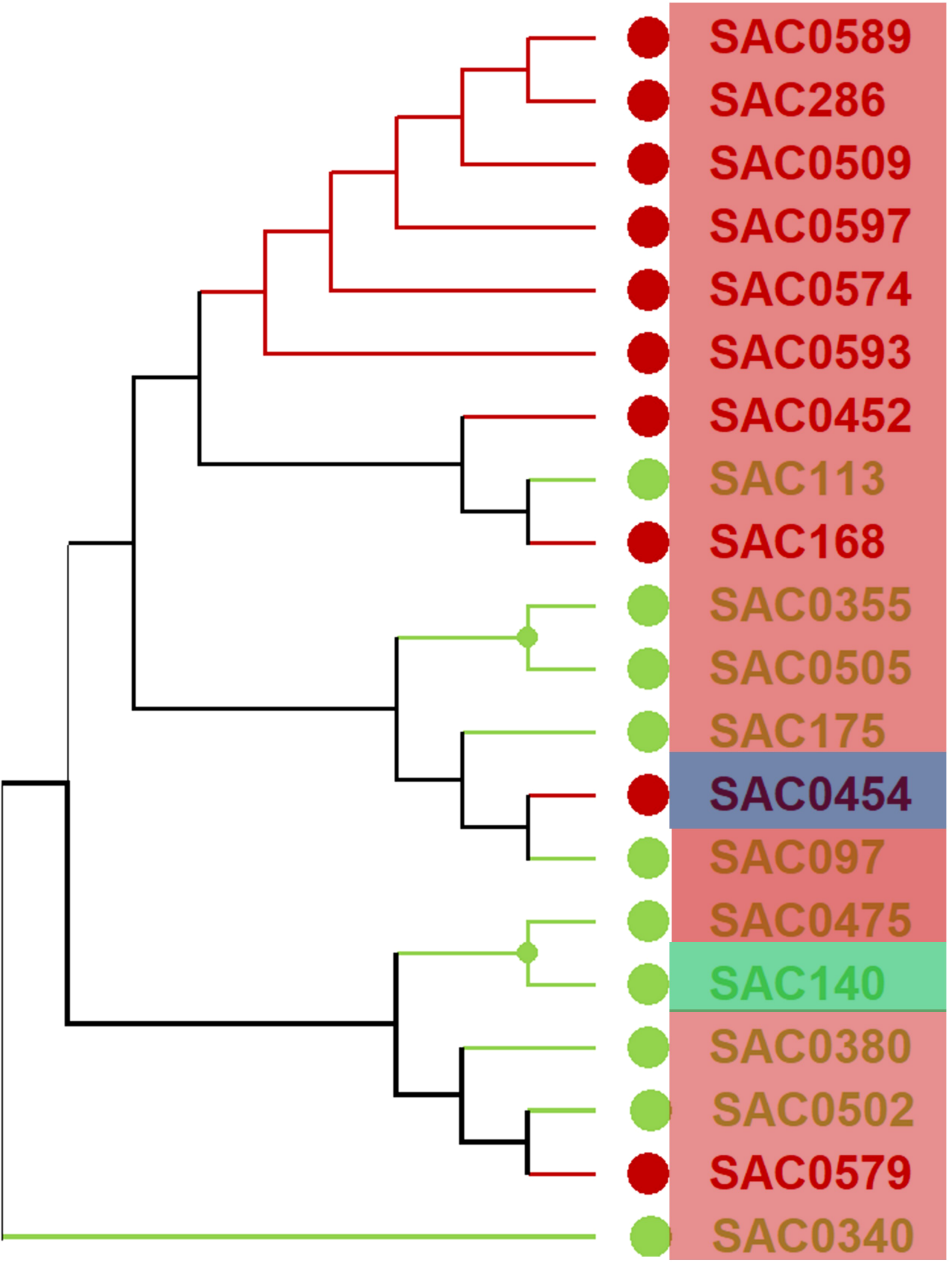
The hierarchical cluster analysis conducted on 20 chosen sugarcane accessions was based on Brix values. The red circles indicate high Brix values, while the green circles refer to low Brix values. The accessions outlined in red boxes correspond to SP1, those in green boxes correspond to SP2, and those in blue boxes correspond to SP3.

Utilizing the selected accessions characterized by high and low Brix values is a key strategy to enhance the genetic diversity of the WCSRG germplasm (**Supplementary Table S6**). An analysis of the genetic distance among these chosen accessions was conducted, and the resultant dendrogram cluster is illustrated in **Figure 8**. The selected genotypes encompass three distinct subpopulations, with genetic distances among these accessions ranging from 0.07 to 0.12. Such low genetic distances suggest a high degree of genomic similarity, likely reflecting a shared breeding history or common genetic background among the accessions. By evaluating the dendrogram cluster, it is possible to identify the most suitable parent plants for sugarcane crossbreeding. The optimal parent combination, as determined by distinct subpopulations, Brix values, and significant genetic distance, is (“SAC0454”, *S. officinarum*, US, SP3 with a Brix of 21.96) crossed with (“SAC0175”, *S. robustum*, SP1, Barbados, with a Brix of 1.13). This crossbreeding has the potential to generate F1 offspring that exhibit high sucrose content inherited from *S. officinarum*, alongside resistance traits conferred by the wild species *S. robustum*, both of which are well-adapted to tropical and subtropical environments.

## Discussion

### Phenotypic variation and trait distributions

A comprehensive phenotypic evaluation of cane quality traits (Brix, Polarity, Moisture, and Fiber) in 397 sugarcane genotypes revealed substantial variability, as evidenced by broad trait ranges, high standard deviations, and distinct distribution patterns within and among species and subpopulations. This variation could potentially be valuable for sugarcane improvement, particularly in breeding programs aimed at enhancing sugar yield and quality. Descriptive statistics indicated that Brix and Polarity exhibited intermediate to high mean values, representing an overall positive sugar content profile of the germplasm. The broad range in the values of Brix measured (1.13-21.96) was also noted by (Aitken et al., 2006), who also noted the same ranges when they performed a survey of various accessions of sugarcane. The broad range measured on Polarity (2.42-83.54) and Fiber (2.22-42.03) agrees with what (Zhou and Gwata, 2015) reported as considerable variation among collections of sugarcane germplasm. Normal distribution of Brix and Polarity indicates that most of the genotypes have moderate to high sugar and therefore are ideal for cane quality traits and other industrial purposes (Jackson, 2005; Srinivasan et al., 2021; Todd et al., 2014). A comparison of trait variation among species reveals Hybrid and *S. robustum* genotypes with high sugar and fiber content and superior agronomic potential. These findings are consistent with previous reports that recognized interspecific hybrids as elite germplasm based on their increased biomass and sucrose yield (Srinivasan et al., 2021). Conversely, species like *S*. *barberi* and *S*. *edule* recorded lower values for sugar-related traits in this study; however, this does not necessarily reflect their overall breeding value, as they may possess unique alleles contributing to stress tolerance, disease resistance, or other agronomic traits not assessed here. Variance analysis indicated that there were significant differences among subpopulations for Polarity and fiber, but not for Brix or moisture. The correlation analysis revealed a moderate positive association between Polarity and Brix, which aligns with their shared involvement in sugar metabolism and accumulation within the stalk. However, the generally weak correlations observed among the other traits suggest complex genetic regulation, likely involving multiple loci with small effects. This observation is consistent with the polygenic nature of quantitative traits in sugarcane, where traits such as sucrose content, fiber, and moisture are influenced by diverse genomic regions, potentially with interacting or pleiotropic effects (Bhatt et al., 2025; Liu P. et al., 2016; Perlo et al., 2020).

### Population structure and genetic differentiation

STRUCTURE analysis of 397 sugarcane genotypes identified three distinct subpopulations (SP1, SP2, SP3), a finding verified by principal component analysis (PCA). Notably, SP1 included most genotypes, indicating a broad genetic base, while SP2 and SP3 were specialized subpopulations. The grouping agrees with the observations in a previous study where sugarcane germplasm exhibited clear genetic stratification by species and countries and institutions was obtained (Xiong et al., 2022). In contrast to the findings of, Saavedra-Díaz et al. (2024) who revealed four subpopulations in 220 genotypes from the Cenicaña’s diverse panel, findings of this study indicate population structure can be very different based on geographical distribution and breeding history. Park et al. (2024) also revealed two distinct subgroups within the *S.* sp*ontaneum* cluster, while the remaining species formed a separate, unified cluster. To examine the substructure within these two clusters, they conducted a STRUCTURE analysis of each cluster using the same procedure and parameters. Hierarchical analysis revealed that two were the most likely number of genetically differentiated groups (K) for the *S.* sp*ontaneum* cluster, and four for the other species.

The highest expected value of heterozygosity was in SP1 at 0.26, revealing a higher rate of genetic diversity than in SP2 at 0.21 and SP3 at 0.11. The genetic diversity of variation among the subpopulations agrees with Wei et al. (2010) who calculated the same heterogeneity rate in sugarcane germplasm. The fixation Index (*Fst*) also reflected the genetic differentiation of the subpopulation. SP2 and SP3 reflected high *F_st_* values of 0.46 and 0.56, respectively, indicating high genetic divergence, whereas SP1 reflected low *F_st_* of 0.06, indicating greater genetic admixture. These results are consistent with the work of Park et al. (2024), which indicated high genetic differentiation among *Saccharum* species, *S. robustum and S. sinense* (*F_st_* = 0.323). Using single-dose SNP marker analysis done by them, the highest mean *F_st_* value was 0.239 in *S. sinense*, followed by 0.224 for *S. robustum*, 0.178 for *S. barberi*, 0.176 for *S.* sp*ontaneum*, and 0.123 for *S. officinarum*, showing various levels of genetic divergence among species. In this study a low level of minor genetic divergence between subpopulations, estimated as 1% difference based on AMOVA, and extensive gene exchange rate (Nm = 29.66) indicate extensive gene exchange at the large scale, as in findings by (You et al., 2016) in evaluating global sugarcane genetic resources. High levels of haploid migrants (Nm = 29.66) confirm the possibility of large-scale gene exchange between subpopulations. Likewise, Park et al. (2024) found that intra-species differences accounted for 85% of the entire genetic diversity and only 15% of the diversity was realized between species.

The allelic variation study within *Saccharum* species revealed contrasting patterns of genetic diversity, emphasizing the species-specific contribution to the sugarcane germplasm pool. *S. robustum*, *S. officinarum*, and *S*. *barberi* possessed the highest value of private alleles, and also high values for unique alleles (Na), effective alleles (Ne), and Shannon’s index of diversity (H), which indicate their broader allelic range and genetic differentiation (Berkman et al., 2014; Xiong et al., 2022). These findings are consistent with previous studies that have highlighted the rich genetic diversity retained in these species, particularly *S*. *robustum*, which has been proposed as a progenitor of cultivated sugarcane and contributes significantly to its genetic base (Wu et al., 2019). In contrast, species such as *S. sinense, S. edule*, and the misidentified group showed minimal allelic diversity and lacked private alleles, likely due to limited sample representation or narrower genetic backgrounds (Wu et al., 2019). Private alleles as well as diversity indices such as Shannon’s index have also proven helpful in the estimation of genetic diversity and identification of unique genetic materials among sugarcane germplasm collections (Xiong et al., 2022). These results are relevant to guiding breeding schemes for expanding the genetic makeup of modern cultivars and maximizing trait introgression across different species. Such information regarding population structure and genetic differentiation will be an important component in breeding, agricultural practice improvement, and in adapting processing technologies for enhancing yield and quality of sugarcane. The genetic structure observed among subpopulations corresponds with known evolutionary relationships: *S. officinarum* is believed to have evolved from *S. robustum*, while *S. sinense* and *S. barberi* are recognized as hybrids of *S. officinarum* and *S.* sp*ontaneum* (Daniels and Roach, 1987). These relationships provide context for the clustering patterns observed in STRUCTURE and PCA analyses.

### Genome wide association mapping for cane traits

Application of three other GWAS models General Linear Model (GLM), Mixed Linear Model (MLM), and Fixed and Random Model Circulating Probability Unification (FarmCPU) is a practically corroborating approach in detecting true associations under confounding effect (Yang et al., 2019). The application of principal components as covariates in GLM effectively circumvented population structure, which was required to avoid false positives in sets of heterogeneous germplasm (Pritchard et al., 2000). The additional incorporation of a kinship matrix accounts for population structure and family relatedness, which further enhances association detection (Yu et al., 2006). The varying number of significant SNPs detected by each model (15 by GLM, 6 by MLM, and 19 by FarmCPU) agrees with previous research demonstrating that various statistical models possess differing stringency and power (Liu P. et al., 2016). MLM will generally call fewer markers due to being more conservative in handling population structure and kinship, thereby lowering false positives but maybe increasing false negatives (Z. Zhang et al., 2010). The enhanced marker provided by the FarmCPU model suggests its increased statistical power employing its unique two-step iterative algorithm with effective confounding effects control but retaining statistical power (Liu X et al., 2016).

The discovery of 21 large-effect markers for Brix on chromosomes 3, 4, 6, and 7 is most interesting. Discovering three Brix markers (AX-171243917-4651, AX-171305424-5045, and AX-171305424-5356) with all three models provides strong evidence for their existence (Tam et al., 2019). Previous studies have placed QTLs for Brix on sugarcane chromosomes 3 and 6 (Singh et al., 2013; Wei et al., 2010). Using the GLM and FarmCPU models, we identified eight markers significantly associated with polarity, located on chromosomes 4, 5, 6, and 8. Notably, the marker AX-171243917–4651 on chromosome 6D was significantly associated with both Brix and polarity, suggesting a potential link to genes involved in sucrose regulation. This is physiological because Polarity has a very close association with sucrose content, which also influences Brix values (Ming et al., 2006). The lack of significant markers for Polarity in the MLM model suggests that this trait may be strongly influenced by population structure and kinship relationships. The comprehensive correction for these factors within the MLM likely reduced spurious associations, resulting in no markers surpassing the significance threshold (Segura et al., 2012). This emphasizes the benefit of having multiple models for the analysis of complex traits in crops with highly complex genetic structures, like sugarcane. The single marker AX−171269335−3422 (chromosome 4D) was consistently detected by all three models for moisture content, indicating a robust and reliable association. Sugarcane moisture content determines processing efficiency and sugar recovery, so this marker would be highly useful in marker-assisted selection (Waclawovsky et al., 2010). The few important markers for this trait indicate that moisture content might be under less complex genetic control than other cane traits or that our marker density was too low to detect all contributing loci.

The identification of eight significant markers associated with fiber content on chromosomes 2, 5, and 7 is very informative regarding the genetic control of this economically important trait. Those two markers (AX-171287224-1157; chr. 2A and AX-171363600-3972; chr. 5D) identified by all three GWAS models to be significantly associated with fiber content, provides high confidence of the validity of these markers warranting further followed up studies of these markers. Previous QTLs for fiber content have been described on chromosomes 2 and 5 (Gouy et al., 2015), consistent with these findings, lending support to the argument that these regions of the chromosome contain genes responsible for cell wall biosynthesis or regulation. To further explore the biological significance of significant SNPs, BLAST searches of their sequences were performed. Several aligned with sugar metabolism–related contigs in public databases (**Supplementary Tables S3**, **S4**).

The multi-model and multi-trait approach employed in this study has also identified several possible markers for the improvement of sugarcane. Most beneficial are the established markers that correspond to more than a single model since these indicate stronger associations less likely to be false positives (Balding, 2006). The three markers for Brix, as well as the two makers for fiber content markers, are strong potential candidates for validation and utilization within marker-assisted selection programs. The fact that AX-171243917-4651; chr. 6D has been found significant for both Brix and Polarity suggests the value of being able to enhance various quality traits parallel, which will enhance breeding efficiency (Chen and Lübberstedt, 2010). While this GWAS analysis identified several encouraging marker-trait associations, the complex polyploid nature of sugarcane presents some unique challenges for genetic analysis (D’Hont et al., 2008). The variation in the number of significant markers identified across models demonstrates the contribution of methodology to GWAS in polyploid crops.

The consistent allelic effects observed in this study align with previous reports of marker-trait associations for sucrose accumulation in sugarcane and related grasses. For example, QTLs for Brix and Pol have been mapped in sugarcane breeding populations (Aitken et al., 2005; Reffay et al., 2005), and several GWAS studies have also identified SNPs linked to sucrose content and fiber traits (Fickett et al., 2019; Xiong et al., 2023; Yang et al., 2019). Similarly, studies in sorghum and maize have revealed allelic variants in sucrose metabolism and transport genes that explain significant variation in sugar accumulation (Chhabra et al., 2021; Liu et al., 2014). Our findings therefore provide complementary evidence that the identified here may represent reliable candidates for marker-assisted selection, even though replication across environments will be required to validate their predictive utility in breeding programs. These findings, though preliminary, suggest possible application in genomic selection schemes, where identified SNPs could serve as predictors for cane quality traits. Future work in replicated trials will be essential to validate and refine these associations.

The recommendation of this study that following experiments will have to utilize a larger number of SNPs marker sets, larger population sizes, and incorporation of haplotype-based approaches to further define the genetic structure of these complex traits (Garcia et al., 2013). Also, the functional characterization of these markers through molecular mapping, gene expression, and ultimately transformation experiments will be necessary to translate these statistical associations into biologically meaningful data (Huang et al., 2010). We acknowledge that the absence of replication and multi-year data limits the robustness of the marker-trait associations. Therefore, the markers identified in this study should be considered preliminary and require validation in replicated, multi-location breeding trials or segregating populations. Although fiber and sucrose are traits typically requiring multi-year, multi-location testing, this study provides a first exploratory analysis across 397 diverse accessions. Future work will evaluate these traits under replicated, multi-year trials to confirm the stability and breeding utility of the associated markers.

## Conclusion

This study offers an insight into genetic diversity and marker-trait associations in traits of sugarcane, with tools available for marker-assisted selection. The identification of stable and pleiotropic SNP markers, particularly for Brix, Polarity and fiber content, indicates their potential utility in improving more than one trait simultaneously. Adequate control of population structure and kinship in the GWAS models rendered the associations more robust. The phenotypic variation seen among *Saccharum* species and the genetic admixture underscore the importance of wide-based germplasm in breeding. However, the polyploid character of sugarcane demands further verification using fine mapping, gene expression, and multi-location testing. Future efforts need to increase marker density, increase population size, and follow haplotype-based and functional genomic approaches to fully value and utilize the genetic basis of cane traits for crop improvement.

## Data Availability

The datasets presented in this study can be found in online repositories. The names of the repository/repositories and accession number(s) can be found in the article/**Supplementary Material**.

## Supplementary material

Supplementary Table 1 The list of 397 sugarcane accessions, species, STURCTURE subpopulation and the mean of 4 cane quality traits.

Supplementary Table 2 The sequences of each marker of 357 SNPs used in this study.

Supplementary Table 3 Detailed GWAS results for the 4 cane quality traits (Brix, polarity, moisture and fiber) using GLM, MLM and FarmCPU model with 357 SNPs.

Supplementary Table 4 List of Common significant markers associated with cane quality traits (Brix, polarity, moisture and fiber) and identified using 3 different GWAS models.

Supplementary Table 5 The highest and lowest ten sugarcane accessions with brix values across homozygous alleles (blue cells) and heterozygous alleles (red cells).

Supplementary Table 6 Pairwise genetic distance among 20 selected sugarcane accessions with extreme Brix values (10 highest and 10 lowest) based on 357 SNP markers.
